# Analysis of sedative-hypnotic drug use trends in children and adolescents (2018–2023): a study based on outpatient prescription data from a general hospital

**DOI:** 10.3389/fphar.2025.1563580

**Published:** 2025-04-17

**Authors:** Zhang Qingyu, Jiang Wenqing, Ma Le, Hou Yanbin, Miao Pingping, Lin Chen, Mao Jiaxin, Dai Ni, Yang Dalu, Tong Kanzhen, Su Junting, Zhu Zhenzhen, Ruan Liemin, Ji Yunxin

**Affiliations:** ^1^ Department of Psychiatry and Behavioral Medicine, First Hospital Affiliated to Ningbo University, Ningbo, Zhejiang, China; ^2^ Department of Child and Adolescent Psychiatry, Shanghai Mental Health Center, Shanghai, China; ^3^ School of Medicine, Ningbo University, Ningbo, Zhejiang, China; ^4^ Department of Psychiatry, Kangning Hospital Affiliated to Ningbo University, Ningbo, Zhejiang, China; ^5^ Department of Psychiatry, Ningbo Kangning Hospital, Ningbo, Zhejiang, China

**Keywords:** children and adolescents, sedative-hypnotic drugs, usage trends, prescription analysis, combination therapy

## Abstract

**Objective:**

This study aims to analyze the use of sedative-hypnotic drugs among children and adolescents in a hospital setting, providing a reference for optimizing drug use.

**Methods:**

A retrospective analysis was conducted on the prescription data of sedative-hypnotic drugs for children and adolescents aged 6–18 years from 2018 to 2023 at the outpatient department of the hospital. Data were organized using Excel and analyzed using statistical software such as SPSS, with descriptive statistics and independent samples t-tests used to analyze medication patterns across different age groups and genders.

**Results:**

The majority of prescriptions originated from the psychiatry department. The most common diagnoses included depressive state, anxiety state, and sleep disorders. Combination therapy with benzodiazepines and antidepressants was the most common treatment regimen. The number of prescriptions showed a yearly increasing trend, rising from 160 in 2018 to 1,583 in 2023, and the total usage also increased annually, from 30.47 g in 2018 to 260.15 g in 2023. Despite the increase in total usage, the drug usage per prescription decreased year by year. Most patients had a medication duration of less than 30 days, and the *per capita* usage increased with longer medication durations. Lorazepam and zopiclone were the most frequently used drugs. The study also found that the duration of medication use was significantly longer in female patients than in males, and significantly longer in the 6–12 age group compared to the 13–18 age group.

**Conclusion:**

The use of sedative-hypnotic drugs in children and adolescents has shown a yearly increasing trend, and the management of sedative-hypnotic drug use in children and adolescents should be strengthened.

## 1 Introduction

In recent years, mental health issues such as depression, anxiety, and sleep disorders have become increasingly prevalent among children and adolescents, resulting in a growing demand for pharmacological interventions. Sedative-hypnotic drugs, as one of the primary treatment options, have been widely used in the management of these mental health conditions. given the distinct physiological and psychological characteristics of children and adolescents compared to adults, this population may exhibit increased sensitivity to drug-related side effects. Therefore, the safety and efficacy of sedative-hypnotic drugs in this demographic require increased clinical attention (Bushnell et al., 2024; Liu et al., 2019).

Sedative-hypnotic drugs primarily include barbiturates, benzodiazepines (BZDs), and Z-drugs (novel sleep-promoting agents). BZDs, due to their potent sedative and hypnotic effects, are widely used in the treatment of anxiety and insomnia. However, prolonged use of these drugs can lead to the development of tolerance, dependence, and withdrawal symptoms, particularly in children and adolescents whose physiological systems are still developing. Therefore, their use in this population requires heightened caution. In contrast, Z-drugs are increasingly preferred as alternatives to BZDs due to their more favorable side effect profile and lower risk of dependence.

The use of sedative-hypnotic drugs in children and adolescents demands careful consideration, as these medications are associated with long-term side effects such as drowsiness, memory impairment, cognitive developmental delays, and an elevated risk of respiratory depression and addiction. However, the importance of their judicious clinical use is crucial, especially in cases of severe sleep disturbances or anxiety symptoms, where short-term, appropriately dosed use can significantly enhance the quality of life for children and adolescents. Supported by real-world clinical data, the judicious use of sedative-hypnotic drugs, when administered under professional guidance, can effectively alleviate symptoms while systematically monitoring and mitigating adverse effects, ensuring children and adolescents achieve optimal therapeutic outcomes.

To the best of our knowledge, no nationwide empirical study has been conducted on the use of sedative-hypnotic drugs in children and adolescents in China. To address this gap and better guide the rational use of sedative-hypnotic drugs in clinical practice, this study investigates the use of these medications in children and adolescents at a tertiary care hospital. The primary objectives are to assess the use of benzodiazepines (BZDs) among this population and provide evidence-based insights for optimizing the use of sedative-hypnotic drugs in children and adolescents.

## 2 Materials and methods

### 2.1 Study subjects

**FIGURE 1:**
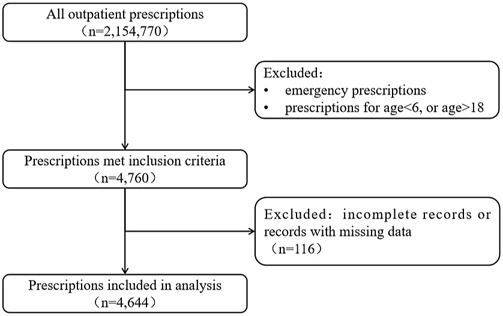
Flow chart.

This study extracted data on sedative-hypnotic drug prescriptions for children and adolescents aged 6–18 years from the rational drug use system of our hospital covering 1 January 2018, and 31 December 2023. The sedative-hypnotic drugs included benzodiazepines (BZDs) and Z-drugs. The exclusion criteria comprised emergency prescriptions and prescriptions for patients outside the 6 to 18 age range, resulting in 4,760 prescriptions. Subsequently, 116 prescriptions with incomplete or missing data were excluded, yielding a final sample of 4,644 valid prescriptions for analysis. Each prescription documented medication use during a single outpatient visit, as illustrated in Figure 1.

### 2.2 Research methods

(1) Prescription Count: The number of prescriptions was counted as individual units, without considering the dosage specified in each prescription.
(2) Drug Utilization: The usage of each sedative-hypnotic drug was standardized by converting it to an equivalent dose of diazepam. The conversion factors for sedative-hypnotic drugs are provided in Table 1 (Brandt et al., 2018).
(3) Average Usage per Patient = Total Usage/Number of Patients
(4) Drug Usage per Prescription = Total Usage/Number of Prescriptions
(5) Defined Daily Dose (DDD): The standard defined by the World Health Organization (WHO), representing the average maintenance dose per day for the drug’s main therapeutic purpose.
(6) Defined Daily Dose System (DDDs): DDDs = Total drug usage over a certain period/The drug’s defined daily dose, with higher DDD values indicating higher frequency of drug use.
(7) Duration of Medication Use: The duration of medication use is determined by cumulatively calculating the total number of days of medication use across all prescriptions for a patient. For each prescription, the number of medication days is calculated by dividing the total amount of medication prescribed by the daily dosage. If the interval between two consecutive prescriptions exceeds 1 month, the calculation is restarted.

**TABLE 1:** Equivalent dose conversion for sedative-hypnotic drugs.

Drug	Equivalent dose (mg)
Diazepam	10
Clonazepam	0.5
Lorazepam	1
Oxazepam	15
Alprazolam	0.5
Estazolam	1.0–2.0
Zolpidem	20
Zopiclone	15

### 2.3 Statistical methods

In this study, data entry and organization were performed using Excel. Subsequently, the collected data were analyzed in detail using statistical software such as SPSS. Data analysis primarily employed descriptive statistics and independent samples t-tests to compare medication usage across different age groups and genders. Prior to performing independent samples t-tests, normality and homogeneity of variance were assessed to ensure that the assumptions were met. The level of statistical significance was set at *p* < 0.05.

### 2.4 Ethical approval

This study was approved by the Ethics Committee of the First Affiliated Hospital of Ningbo University (Approval No.: 2025-052RS) and registered with the National Health Security Information Platform (Registration No.: MR-33-25–014962). Since this is a retrospective study and all data were anonymized, the Ethics Committee waived the requirement for written informed consent from patients in accordance with national laws and regulations (Ethical Review Measures for Biomedical Research Involving Humans in China). All patient information was kept strictly confidential throughout the analysis. No identifiable patient information was collected, stored, or analyzed, and strict confidentiality measures were implemented to ensure compliance with data privacy and security protocols.

## 3 Results

### 3.1 Analysis of prescription patterns and combination therapy

As shown in Table 2, among the 4,644 valid prescriptions, female patients accounted for a significantly higher proportion (3,400 cases, 73.42%) than male patients (1,244 cases, 26.58%), resulting in a female-to-male ratio of approximately 2.73:1. Most patients were aged 13–18 years (4,511 cases, 97.14%), with a mean age of 15.86 ± 1.73 years. Regarding prescription sources, most prescriptions originated from the psychiatry department (3,736 prescriptions, 80.28%), followed by the sleep medicine department (508 prescriptions, 10.92%) and the neurology department (171 prescriptions, 3.67%).

**TABLE 2:** Prescription characteristics and analysis of combination therapy.

Category	Number (n)	Percentage (%)
Gender		
Female	3,400	73.42
Male	1,244	26.58
Age		
6–12 years	133	2.86
13–18 years	4,511	97.14
Prescription Source		
Psychiatry Department	3,736	80.28
Sleep Medicine Department	508	10.92
Neurology Department	171	3.67
Other Departments	239	5.13
Diagnosis		
Single Diagnosis	2,790	60.08
Second Diagnosis	1,581	34.04
Third Diagnosis	273	5.88
Top 10 First Diagnoses		
Depressive State	1,250	26.93
Anxiety State	845	18.21
Sleep Disorders	487	10.49
Depressive Episode	477	10.28
Bipolar Disorder	236	5.09
Anxiety-Depressive State	226	4.87
Moderate Depressive Episode with Somatic Symptoms	98	2.11
Obsessive State	88	1.90
Severe Depressive Episode without Psychotic Symptoms	79	1.70
Epilepsy	63	1.36
Combination Therapy		
Benzodiazepines + Antidepressants	404	54.74
Benzodiazepines + Mood Stabilizers	178	24.12
Benzodiazepines + Antipsychotics	67	9.08
Benzodiazepines + Antiepileptics	55	7.45
Benzodiazepines + Benzodiazepines	22	2.98
Benzodiazepines + Z-drugs	12	1.63

For diagnoses, prescriptions with a single diagnosis accounted for the highest proportion (2,790 prescriptions, 60.08%), while those with two and three diagnoses represented 34.04% (1,581 prescriptions) and 5.88% (273 prescriptions), respectively. Among the top ten primary diagnoses, depressive state (1,250 cases, 26.93%) and anxiety state (845 cases, 18.21%) were most common, followed by sleep disorders (487 cases, 10.49%) and depressive episodes (477 cases, 10.28%).

For combination therapy, the most common regimen was the combination of benzodiazepines with antidepressants (404 cases, 54.74%), followed by benzodiazepines with mood stabilizers (178 cases, 24.12%). Combinations of benzodiazepines with antipsychotics (67 cases, 9.08%) and antiepileptics (55 cases, 7.45%) were also relatively frequent. In contrast, combinations of benzodiazepines with other benzodiazepines (22 cases, 2.98%) and with Z-drugs (12 cases, 1.63%) were less common (Table 2).

### 3.2 Trends in the usage of sedative-hypnotic drugs

**FIGURE 2:**
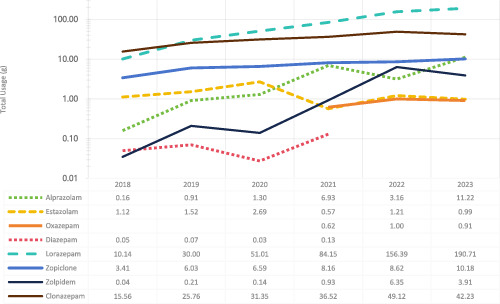
Annual total usage trends of various sedative-hypnotic drugs.

As shown in Table 3, the number of prescriptions increased annually, rising from 160 in 2018 to 1,583 in 2023. During the same period, the number of patients also grew from 103 in 2018 to 830 in 2023. Additionally, total usage showed an upward trend, increasing from 30.47 g in 2018 to 260.15 g in 2023. Specifically, the annual total usage of various sedative-hypnotic drugs displayed different trends (Figure 2). The usage of lorazepam increased steadily, while estazolam and clonazepam showed an initial rise followed by a slight decline. The usage of oxazepam and diazepam remained relatively low with minimal variation.

**TABLE 3:** Annual usage of sedative-hypnotic drugs.

Year	2018	2019	2020	2021	2022	2023
Number of Prescriptions (n)	160	350	542	736	1,273	1,583
Number of Patients (n)	103	179	272	404	555	830
Total Usage (grams)	30.47	64.50	93.10	138.00	225.85	260.15
Mean Usage per Patient (grams/patient)	0.30	0.36	0.34	0.34	0.41	0.31
Usage per Prescription (grams/prescription)	0.19	0.18	0.17	0.19	0.18	0.16

**FIGURE 3:**
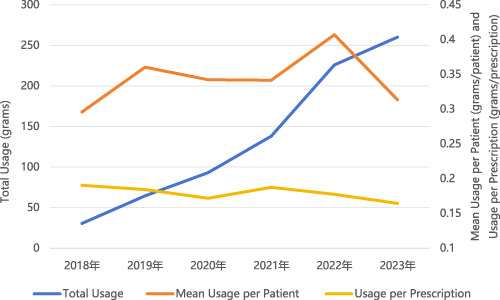
Trends in total usage, mean usage per patient, and usage per prescription of sedative-hypnotic drugs.

Although total usage of sedative-hypnotic drugs increased each year, the drug usage per prescription declined gradually. Specifically, the usage per prescription decreased from 0.19 g in 2018 to 0.16 g in 2023. Meanwhile, the mean usage per patient exhibited fluctuating trends. In 2019 and 2020, the mean usage per patient was 0.36 g and 0.34 g, respectively, showing a slight decrease, followed by an increase to 0.41 g in 2022, before dropping again to 0.31 g in 2023. Detailed data are shown in Table 3 and Figure 3.

### 3.3 Duration of sedative-hypnotic drug use

**FIGURE 4:**
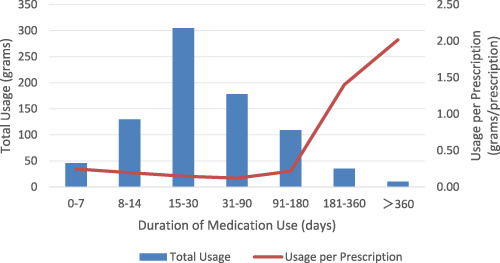
Trends in Drug Usage and Drug Usage per Prescription across varying Durations of Medication Use.

The highest number of prescriptions occurred for the duration of medication use between 15 and 30 days, totaling 2,077 prescriptions, followed by 31–90 days (1,480 prescriptions) and 8–14 days (655 prescriptions). The drug usage per prescription showed an initial decline followed by a subsequent increase. It decreased from 0.25 g per prescription in the 0–7 day period to 0.12 g per prescription in the 31–90 day period. However, as the duration of medication use increased, the drug usage per prescription gradually rose, reaching 0.22 g per prescription in the 91–180 day period, 1.40 g per prescription in the 181–360 day period, and 2.02 g per prescription in the >360 day period (Table 4; Figure 4).

**TABLE 4:** Number of Prescriptions and Total Drug Usage across varying Durations of Medication Use.

Duration of medication use (days)	0–7	8–14	15–30	31–90	91–180	181–360	>360
Number of Prescriptions (n)	185	665	2077	1,480	507	25	5
Total Usage (grams)	45.68	129.47	304.5	178.22	109.11	35.01	10.08
Usage per Prescription (grams/prescription)	0.25	0.19	0.15	0.12	0.22	1.40	2.02

### 3.4 DDDs and ranking of sedative-hypnotic drugs

Lorazepam and Zopiclone consistently ranked first and second, demonstrating their widespread clinical use and high usage frequency. The usage frequency of Zolpidem and Alprazolam has increased annually. Oxazepam, which started being used in 2021, has shown an upward trend in usage frequency, although its overall usage remains low. The usage frequency of Clonazepam and Estazolam has gradually declined, indicating a decrease in their clinical use. Diazepam and Phenobarbital have consistently ranked at the bottom in terms of usage frequency. The specific data are presented in Table 5.

**TABLE 5:** DDDs of sedative-hypnotic drugs.

Drug	2018		2019		2020		2021		2022		2023	
	DDDs	Ranking	DDDs	Ranking	DDDs	Ranking	DDDs	Ranking	DDDs	Ranking	DDDs	Ranking
Alprazolam	32.00	6	53.60	6	70.40	5	375.60	3	278.00	5	644.20	4
Estazolam	56.00	4	89.33	4	134.67	4	45.00	7	75.33	7	177.33	6
Oxazepam							18.00	8	40.20	8	102.00	7
Diazepam	5.00	8	7.00	8	2.75	8	13.00	9				
Phenobarbital	40.00	5	20.00	7	36.00	6	144.40	6	106.00	6	20.00	8
Lorazepam	606.60	1	1,519.80	1	2,528.20	1	4,517.60	1	6,739.80	1	8,489.40	1
Zopiclone	562.00	2	951.00	2	1,139.00	2	1,431.00	2	1,565.00	2	1708.00	2
Zolpidem	14.00	7	56.00	5	35.00	7	217.00	5	1,103.50	3	735.00	3
Clonazepam	94.25	3	161.00	3	195.00	3	227.00	4	303.75	4	260.00	5

### 3.5 Gender and age

As shown in Table 6, there were statistically significant differences between genders in both the duration of medication use and DDDs (*P* < 0.05). Additionally, statistically significant differences were observed in the usage of lorazepam and clonazepam (*P* < 0.05).

**TABLE 6:** Analysis of usage by gender.

Category	Gender		T	P
	Male	Female		
Number of Prescriptions (n)	1,244	3,400		
Number of Patients (n)	629	1,453		
Total Usage (grams)	227.37	584.70		
Usage (grams)	0.36 ± 1.04	0.40 ± 1.89	−1.51	0.61
Usage per Prescription (grams/prescription)	0.18 ± 0.28	0.17 ± 0.28	−1.15	0.25
Duration of Medication Use (days)	38.64 ± 63.42	45.81 ± 87.84	−2.32	0.02
DDDs	7.67 ± 6.26	6.64 ± 5.15	5.18	0.001
Usage by Drug (grams)				
Alprazolam	0.16 ± 0.09	0.16 ± 0.08	−2.23	0.82
Estazolam	0.09 ± 0.05	0.09 ± 0.06	−0.10	0.92
Oxazepam	0.38 ± 0.00	0.36 ± 0.14	1.77	0.87
Diazepam	0.12 ± 0.00	0.13 ± 0.01	−0.04	0.97
Lorazepam	0.15 ± 0.15	0.14 ± 0.10	2.63	0.01
Clonazepam	1.10 ± 7.80	1.59 ± 0.87	−3.41	0.001
Zopiclone	0.10 ± 0.05	0.11 ± 0.07	−1.87	0.06
Zolpidem	0.08 ± 0.06	0.07 ± 0.05	0.20	0.84

As shown in Table 7, there were statistically significant differences in the duration of medication use among different age groups (*P* < 0.05). Additionally, statistically significant differences were observed in the usage of clonazepam (*P* < 0.05)

**TABLE 7:** Analysis of usage by age group.

Category	Age group		T	P
	6–12	13–18		
Number of Prescriptions (n)	133	4,511		
Number of Patients (n)	71	2,011		
Total Usage (grams)	31.42	780.65		
Usage (grams)	0.44 ± 0.83	0.39 ± 1.70	0.27	0.79
Usage per Prescription (grams/prescription)	0.24 ± 0.43	0.17 ± 0.28	1.68	0.10
Duration of Medication Use (days)	23.40 ± 9.06	19.53 ± 6.44	2.79	0.008
DDDs	5.33 ± 6.66	6.94 ± 5.48	−1.58	0.06
Usage by Drug (grams)				
Alprazolam	0.12 ± 0.08	0.16 ± 0.08	−1.29	0.20
Estazolam	0.07 ± 0.03	0.09 ± 0.04	−0.68	0.50
Diazepam	0.01 ± 0.02	0.01 ± 0.01	−0.91	0.37
Lorazepam	0.15 ± 0.13	0.14 ± 0.10	0.89	0.37
Clonazepam	0.64 ± 0.89	1.56 ± 0.79	−5.11	0.001

## 4 Discussion

### 4.1 Prescription sources and combination therapy

Regarding the sources of prescriptions, this study found that the vast majority of sedative-hypnotic drug prescriptions originated from psychiatry (80.28%), with a relatively lower proportion from non-psychiatric sources. Prescriptions issued by psychiatry departments help ensure the rational and safe use of these drugs, thereby reducing the risk of misuse and dependence. However, the data in this study are limited to outpatient patients from a single hospital and only cover children and adolescents aged 6–18 years, which may restrict the generalizability of the findings to broader populations. Currently, large-scale investigations into the sources of sedative-hypnotic drug prescriptions are scarce in China. In contrast, a nationwide study in Sweden analyzed the prescriptions and usage of benzodiazepines among individuals aged 0–24 years from 2006 to 2013, revealing that nearly 65% of initial BZD prescriptions originated from non-psychiatric healthcare providers, typically from primary care or other non-psychiatric specialties (Sidorchuk et al., 2018). Given that prescriptions from non-psychiatric departments may affect the rational use and safety of these drugs, it is recommended to strengthen the regulation of BZD prescriptions, particularly in non-psychiatric settings. Furthermore, there is an urgent need to conduct large-scale nationwide studies to comprehensively evaluate prescription patterns across different departments, ensuring that children, adolescents, and young adults receive safe and effective treatment with sedative-hypnotic drugs.

In terms of comorbid diagnoses and combination therapy, this study found that depressive state, anxiety state, and sleep disorders were the most common diagnoses. More than half of the combination therapies involved antidepressants, followed by mood stabilizers and antipsychotics. Recent research have found that nearly three-quarters of young patients with insomnia also have a psychiatric diagnosis (McCall, 2024). Additionally, over 75% of patients with insomnia concurrently used other psychotropic medications, including 49.58% using antidepressants, 14.68% using antipsychotics, and 38.76% using other anxiolytics or sedative-hypnotics (Antoniou et al., 2023). Consistent with these findings, a Swedish study also indicated that more than 75% of patients using BZDs were concurrently prescribed other psychotropic medications, particularly antidepressants (46%) (Sidorchuk et al., 2018). In clinical practice, emotional and sleep problems often coexist, making the combined use of sedative-hypnotics and antidepressants quite common. These findings highlight the importance of recognizing and addressing emotional issues in the treatment of children and adolescents, ensuring comprehensive and safe therapeutic approaches.

### 4.2 Usage and duration of medication use of sedative-hypnotic drugs

This study found that the total usage of sedative-hypnotic drugs has increased annually. This trend may be related to the rising prevalence of emotional and sleep problems in recent years, which has led to an increase in the number of patients seeking medical attention, thereby driving the upward trend in total usage. Additionally, the expansion of hospital capacity, improvements in diagnostic and treatment capabilities, increased social stress, and growing public awareness of mental health issues may have also contributed to this change. Despite the continuous increase in total usage, the drug usage per prescription showed a downward trend, dropping from 0.19 g/prescription in 2018 to 0.16 g/prescription in 2023, which may be attributed to the increasing standardization of sedative-hypnotic drug use by hospitals and physicians. Furthermore, the trends in usage varied among different sedative-hypnotic drugs, with the usage of alprazolam and lorazepam increasing significantly, likely due to their reliable effects and low treatment costs, leading to widespread clinical use.

Over the past 20 years, the prescription rates of BZDs for children and adolescents have increased in many Western countries (Alessi-Severini et al., 2014; Carrasco-Garrido et al., 2024; Huerta et al., 2016; Sidorchuk et al., 2018). A domestic study analyzed the prescription trends of psychotropic drugs in urban areas of China from 2013 to 2017, revealing a significant increase in the prescription rate of sedative-hypnotic drugs, from 3.17% in 2013 to 5.38% in 2017, an increase of 69.61% (Xu et al., 2021). However, some studies have found that the prescription rates of BZDs for individuals under 18 are relatively low and show a decreasing trend (Bénard-Laribière et al., 2017; Cheng et al., 2021). Currently, data on the usage rates and patterns of sedative-hypnotic prescriptions mainly come from adults and the elderly, while evidence regarding the use of these drugs in younger populations is limited, likely because such medications are generally not recommended for pharmacological treatment in individuals under 18 years of age (Riemann et al., 2023; Society, 2017).

The number of prescriptions reached its peak for the Duration of Medication Use of 15–30 days (2,077), followed by 31–90 days (1,480) and 8–14 days (655), suggesting clinicians prefer short-to-medium term sedative-hypnotic regimens. Notably, the Drug Usage per Prescription exhibited a significant increasing trend when the Duration of Medication Use exceeded 180 days, a phenomenon potentially associated with the complexity of conditions in long-term users and the development of drug tolerance. Although this study did not conduct an in-depth analysis of the underlying causes, existing literature suggests that prolonged use of sedative-hypnotics, particularly in adolescent populations, may have potential implications for cognitive function and the risk of drug dependence (Crowe and Stranks, 2018; Olfson et al., 2015). Therefore, for patients requiring long-term medication, clinicians should rigorously evaluate the necessity of continued use and enhance monitoring protocols to minimize potential risks.

### 4.3 Usage frequency of sedative-hypnotic drugs

The usage frequency of zolpidem and alprazolam has increased annually, especially Zolpidem, whose DDDs rose from 14.00 in 2018 to 735.00 in 2023. A study in Zhejiang Province showed that from 2011 to 2016, the usage and sales of sedative-hypnotic drugs exhibited an overall increasing trend, with zolpidem tablets ranking first in sales for six consecutive years (Ding et al., 2019). This suggests that despite the higher treatment costs of new sedative-hypnotic drugs such as zolpidem and zopiclone tablets, their shorter half-lives may result in less residual drug effects on cognitive function the next day, a lower risk of drug dependence compared to BZDs, and no significant adverse effects with long-term use, leading to their widespread clinical application. On the other hand, BZDs remain a common empirical choice for physicians due to their reliable efficacy, high selectivity, broad indications, wide safety margin, and low cost. For example, lorazepam and zopiclone have consistently ranked in the top two positions in terms of usage frequency and ranking ratio, reflecting their widespread clinical use and high usage frequency. In contrast, diazepam has lower DDDs and a lower ranking. likely due to its long half-life and higher incidence of adverse effects, which may contribute to its declining usage frequency.

### 4.4 Gender and age

This study found that the duration of medication use was significantly longer in female patients than in male patients, which may increase the risk of drug dependence and misuse among females. Similar findings have been reported in other studies. One study noted that the probability of benzodiazepine and Z-drug misuse was significantly higher among female adolescents compared to males (Carrasco-Garrido et al., 2024). Additionally, a Norwegian study analyzed the prescription trends of sedative-hypnotic drugs among adolescents from 2012 to 2020, showing that drug use was significantly higher among female adolescents during this period, with the largest increase observed between 2016 and 2018 (Lien et al., 2023). Furthermore, a study from New Zealand found that the use of sedative-hypnotic drugs was higher among females and adolescents from low-income families between 2008 and 2016, with an increasing trend [2]. These findings suggest that gender plays a significant role in drug use and misuse, highlighting the need for special attention to drug dependence in female patients in clinical practice and the implementation of appropriate interventions.

This study also found that the proportion of prescriptions for the 6–12 age group was relatively low, indicating relatively standardized use of sedative-hypnotic drugs in this age group in clinical practice. However, it is noteworthy that the duration of medication use was significantly longer in the 6–12 age group compared to the 13–18 age group. This difference may be related to the types of diseases prevalent in this age group. However, this study did not analyze the specific diagnoses and medication patterns in this group in detail, and future research should focus on the types of drugs used and their indications in this age group to further understand the underlying reasons for medication use. Given that physiological development is incomplete in younger age groups, non-pharmacological treatments should be the first-line approach, while pharmacological treatments should be used cautiously under clear indications to avoid the risk of long-term drug dependence (Liu et al., 2019.

### 4.5 Challenges and difficulties

In recent years, the mental health status of children and adolescents in China has raised significant concerns, with high prevalence rates of depression and sleep disorders. For example, relevant studies indicate that 14.8% of adolescents may exhibit some degree of depressive symptoms (Fu et al., 2023). Among children aged 5–12 years, approximately one-fifth experience insomnia, and the incidence rate has been increasing annually (Himelfarb and Shatkin, 2024). Due to the severity of these issues, there has been a noticeable trend toward younger patients in psychiatric consultations in recent years, with the use of psychotropic drugs among children and adolescents becoming increasingly common. A domestic study analyzed the usage rates and patterns of psychotropic drugs among Chinese children using cross-sectional data from the national basic medical insurance database. Showing that the usage rate of sedative-hypnotic drugs was the highest, at 11.8‰, significantly higher than other categories of psychotropic drugs (Zhang et al., 2023). However, current domestic and international guidelines impose strict age restrictions on the use of sedative-hypnotic drugs in children and adolescents. For example, the United States has not approved any drugs specifically for treating insomnia in children under 16 years old, and most drugs used to treat adult insomnia are not recommended for children (Nunes and Bruni, 2015; Society, 2017; Su and Lu, 2018). There is currently a lack of standards or guidelines for the use of sedative-hypnotic drugs in children and adolescents, and related research is very limited. There is insufficient evidence-based support for the efficacy, safety, and tolerability of insomnia medication in children and adolescents, with most practices relying on clinical experience Lu, 2019) Therefore, after assessing the benefits and risks, psychiatrists in clinical practice are often compelled to use sedative-hypnotic drugs for some patients, which frequently involves off-label or non-guideline-compliant use.

To address this pressing issue, a multi-faceted approach is essential to tackle the challenges and difficulties associated with the use of sedative-hypnotic drugs in children and adolescents. Firstly, healthcare professionals should strictly follow relevant guidelines and standards, ensuring clear indications for drug use and avoiding unnecessary pharmacological treatments. Whenever possible, non-pharmacological treatment options, such as cognitive-behavioral therapy (CBT), sleep hygiene education, and relaxation training, should be prioritized (Society, 2017). Before initiating sedative-hypnotic drug therapy, a comprehensive risk assessment should be conducted, carefully evaluating the potential risks and benefits of the medication. During treatment, the patient’s response to the drug and any adverse effects should be closely monitored, and treatment plans should be adjusted promptly as needed. Secondly, hospitals should robust comprehensive drug use management protocols and regulatory mechanisms, strengthening training and education to enhance the expertise and skills of psychiatrists and other relevant healthcare professionals, ensuring that all prescribing and medication practices comply with standards and regulations. Additionally, scientific research on the use of sedative-hypnotic drugs in children and adolescents should be promoted to accumulate more evidence to support clinical decision-making and to drive updates and improvements to relevant guidelines. Finally, the government should develop and refine relevant policies, and public awareness campaigns should be conducted to increase the importance placed on the mental health and rational drug use of children and adolescents throughout society.

## 5 Limitation

It is important to note that although our study reflects the medication trends in our hospital, the results may not be widely generalizable to other regions or larger populations due to limitations in sample size and geographic scope. In particular, the hospital-based patient population may have selection biases, and the medication patterns may differ from national trends or epidemiological patterns in other regions. Additionally, the study only analyzed prescription data and did not include dispensing data, which may limit the ability to fully reflect actual medication usage. Moreover, drug shortages could have influenced prescribing trends, and this factor should be considered as a limitation of the study. Therefore, future studies should expand to multicenter and multiregional samples, incorporating dispensing data to further evaluate the use of sedative-hypnotic drugs in different contexts, thereby providing more representative and widely applicable evidence.

## 6 Conclusion

This study found that, over the past 5 years, the use of sedative-hypnotic drugs among children and adolescents in our hospital has shown a year-on-year increase, with a significant rise in both the number of prescriptions and total drug consumption. The combination of benzodiazepines and antidepressants was commonly prescribed. Despite the overall increase in drug usage, the per-prescription drug consumption has decreased each year, and most patients used the medication for less than 30 days. However, as the duration of use increased, the *per capita* consumption significantly rose. Lorazepam and zopiclone were the most commonly used drugs. Additionally, the study found that female patients had significantly longer medication durations compared to male patients, and children aged 6–12 had longer medication durations than those aged 13–18. Given the limited sample size, it is necessary to conduct further studies with larger sample sizes, and future efforts should focus on strengthening the management of sedative-hypnotic drug use in children and adolescents.

## Data Availability

Data supporting this study are available upon reasonable request from the corresponding authors, subject to ethical approvals and data privacy regulations. The raw patient data cannot be publicly shared due to confidentiality restrictions imposed by the Ethics Committee of the First Affiliated Hospital of Ningbo University.

## References

[B1] Alessi-Severini S. Bolton J. M. Enns M. W. Dahl M. Collins D. M. Chateau D. (2014). Use of benzodiazepines and related drugs in manitoba: a population-based study. Cmaj Open 2, E208–E216. 10.9778/cmajo.20130076 PMC4251517 25485245

[B2] Antoniou T. Mccormack D. Kitchen S. Pajer K. Gardner W. Lunsky Y. (2023). Impact of a publicly-funded pharmacare program policy on benzodiazepine dispensing among children and youth: a population-based natural experiment. Bmc Pediatr. 23, 519. 10.1186/s12887-023-04331-4 37858122 PMC10585894

[B3] Bénard-Laribière A. Noize P. Pambrun E. Bazin F. Verdoux H. Tournier M. (2017). Trends in incident use of benzodiazepines and z-drugs in France from 2006 to 2012: a population-based study. Pharmacoepidemiol. Drug Saf. 1467–1475. 10.1002/pds.4296 27807907

[B4] Brandt J. Alkabanni W. Alessi-Severini S. Leong C. (2018). Translating benzodiazepine utilization data into meaningful population exposure: integration of two metrics for improved reporting. Clin. Drug Investig. 38, 565–572. 10.1007/s40261-018-0648-y 29619753

[B5] Bushnell G. A. Rynn M. A. Gerhard T. Keyes K. M. Hasin D. S. Cerdá M. (2024). Drug overdose risk with benzodiazepine treatment in young adults: comparative analysis in privately and publicly insured individuals. Addiction 119, 356–368. 10.1111/add.16359 37816665 PMC10838605

[B6] Carrasco-Garrido P. Hernández-Barrera V. Jiménez-Trujillo I. Lima F. L. Gallardo P. C. Yeamans S. (2024). Trends in the nonmedical misuse of benzodiazepines and z-hypnotics among school-aged adolescents (2016-2021): gender differences and related factors. Child. Adolesc. Ment. Health 29, 345–354. 10.1111/camh.12716 38778447

[B7] Cheng S. Sun H. H C. (2021). Prescription patterns and trends of anxiolytics and hypnotics/sedatives among child and adolescent patients with psychiatric illnesses in a psychiatric center of northern taiwan. Taiwan. J. Psychiatry 35, 82–89. 10.4103/tpsy.tpsy_18_21

[B8] Crowe S. F. Stranks E. K. (2018). The residual medium and long-term cognitive effects of benzodiazepine use: an updated meta-analysis. Arch. Clin. Neuropsychol. 33, 901–911. 10.1093/arclin/acx120 29244060

[B9] Ding H. Wang J. Wu F. (2019). Analysis of sedative-hypnotic drug use in 11 hospitals in Zhejiang Province from 2011 to 2016. Eval. Analysis Drug-Use Hosp. China 19, 466–470. 10.14009/j.issn.1672-2124.2019.04.026

[B10] Fu X. Zhang K. Chen X. Chen Z. (2023). Report on the development of national mental health in China (2021-2022). Social Sciences Academic Press.

[B11] Himelfarb M. Shatkin J. P. (2024). Pediatric insomnia. Psychiatr. Clin. North Am. 47, 121–134. 10.1016/j.psc.2023.06.008 38302201

[B12] Huerta C. Abbing-Karahagopian V. Requena G. Oliva B. Alvarez Y. Gardarsdottir H. (2016). Exposure to benzodiazepines (anxiolytics, hypnotics and related drugs) in seven european electronic healthcare databases: a cross-national descriptive study from the protect-eu project. Pharmacoepidemiol Drug Saf. 25 (Suppl. 1), 56–65. 10.1002/pds.3825 26149383

[B13] Lien L. Bonsaksen T. Holte S. T. Kleppang A. L. Steigen A. M. Leonhardt M. (2023). Time trends in self-reported depressive symptoms, prescription of antidepressants, sedatives and hypnotics and the emergence of social media among Norwegian adolescents. Plos One 18, e295384. 10.1371/journal.pone.0295384 PMC10752533 38150420

[B14] Liu X. I. Schuette P. Burckart G. J. Green D. J. La J. Burnham J. M. (2019). A comparison of pediatric and adult safety studies for antipsychotic and antidepressant drugs submitted to the United States food and drug administration. J. Pediatr. 208, 236–242. 10.1016/j.jpeds.2018.12.033 30679050 PMC7171692

[B15] Lu L. (2019). Comprehensive prevention and treatment guidelines for insomnia disorders in. China. Beijing: People's Medical Publishing House.

[B16] McCall W. V. (2024). Insomnia medications for children, adolescents, and young adults: shedding light in the darkness. Sleep 47, zsae058. 10.1093/sleep/zsae058 38430554

[B17] Nunes M. L. Bruni O. (2015). Insomnia in childhood and adolescence: clinical aspects, diagnosis, and therapeutic approach. J. Pediatr. Rio J. 91, S26–S35. 10.1016/j.jped.2015.08.006 26392218

[B18] Olfson M. King M. Schoenbaum M. (2015). Benzodiazepine use in the United States. Jama Psychiatry 72, 136–142. 10.1001/jamapsychiatry.2014.1763 25517224

[B19] Riemann D. Espie C. A. Altena E. Arnardottir E. S. Baglioni C. Bassetti C. (2023). The european insomnia guideline: an update on the diagnosis and treatment of insomnia 2023. J. Sleep. Res. 32, e14035. 10.1111/jsr.14035 38016484

[B100] Sidorchuk A. Isomura K. Molero Y. Hellne C. Lichtenstein P. Chang Z. (2018). Benzodiazepine prescribing for children, adolescents, and young adults from 2006 through 2013: a total population register-linkage study. PLOS Med. 15, e1002635. 10.1371/journal.pmed.1002635 PMC6080748 30086134

[B20] Society C. S. R. (2017). Guidelines for the diagnosis and treatment of insomnia in China. Chin. Med. J. Engl. 97, 1844–1856. 10.3760/cma.j.issn.0376-2491.2017.24.002

[B21] Su L. Lu Z. (2018). Interpretation of the 2017 Chinese guidelines for the diagnosis and treatment of insomnia. World Clin. Drugs 39, 217–222. 10.13683/j.wph.2018.04.001

[B22] Xu L. Lv X. Wang H. Liu Q. Zhou S. Gao S. (2021). Trends in psychotropic medication prescriptions in urban China from 2013 to 2017: national population-based study. Front. Psychiatry 12, 727453. 10.3389/fpsyt.2021.727453 34512424 PMC8424045

[B23] Zhang X. Hu X. Zhao Y. Nie X. Shi L. (2023). Analysis of the current status of psychotropic drug use among children in China. Chin. Pharm. J. 58, 933–938. 10.11669/cpj.2023.10.010

